# Citrus Pectin-Derived Carbon Microspheres with Superior Adsorption Ability for Methylene Blue

**DOI:** 10.3390/nano7070161

**Published:** 2017-06-30

**Authors:** Wenlin Zhang, Zhiqin Zhou

**Affiliations:** 1 College of Horticulture and Landscape Architecture, Southwest University, Chongqing 400716, China; zhangwenlin88519@126.com; 2 Institute of Special Plants, Chongqing University of Arts and Sciences, Yongchuan 402160, China; 3 Key Laboratory of Horticulture Science for Southern Mountainous Regions, Ministry of Education, Chongqing 400715, China

**Keywords:** citrus pectin, carbon microspheres, methylene blue, adsorption

## Abstract

In this study, citrus pectin-derived, green, and tunable carbon microspheres with superior adsorption capacity and high adsorption rate, as well as good reusability toward methylene blue adsorption, were prepared by a facile hydrothermal method without any hazardous chemicals. The materials hold great potential for the treatment of methylene blue wastewater.

## 1. Introduction

Synthetic organic dyes have been treated as a class of serious pollutants in surface water due to their wide applications in the textile, leather, paper, and printing industries [1,2]. These dyes could cause environmental problems and are toxic for humans [3]. Thus, it is necessary to remove these dyes from wastewaters. Methylene blue (MB) is a typical organic dye with toxicity, which could be found easily in dye wastewaters [4]. To date, many approaches such as chemical oxidation, adsorption, photocatalysis, and membrane filtration have been employed to remove MB, of which adsorption is considered as the most effective and economical method [5]. It is worth noting that carbon micro/nanospheres are of great interest as adsorbents in MB removal due to their large specific surface area and high chemical stability [6,7]. In order to enhance the adsorption capacity of MB, most carbon spheres are need to be further activated by KOH and ZnCl_2_ under high temperature conditions [8,9]. However, these activation processes involve a high cost or complicated procedures, while the activating agents are hazardous. Therefore, developing low-cost and green carbon spheres with excellent adsorption performance for MB is highly desirable.

Citrus pectin is a natural polysaccharide which consists of (1-4) linked α-d-galacturonic acid backbones with many carboxyl and hydroxyl groups, and has been extensively applied in food and biomedical industries [3,10,11]. Citrus pectin can be easily and largely obtained from citrus pomace, which is a citrus industrial by-product [12]. Noticeably, a huge amount of citrus pomace is commonly discarded as waste, leading to environment problems [12]. For waste utilization, employing citrus pectin as a precursor to synthesize carbon microspheres is considered. To our knowledge, unique carbon microspheres derived from citrus pectin as an adsorbent for MB has not yet been reported. In this work, citrus pectin-derived carbon microspheres (CPCMs) were synthetized by a facile hydrothermal method without any hazardous chemicals, showing superior adsorption capacity, high adsorption rate, and good reusability for MB adsorption, thus holding great potential for treating MB wastewater.

## 2. Results and Discussion

**Figure 1:**
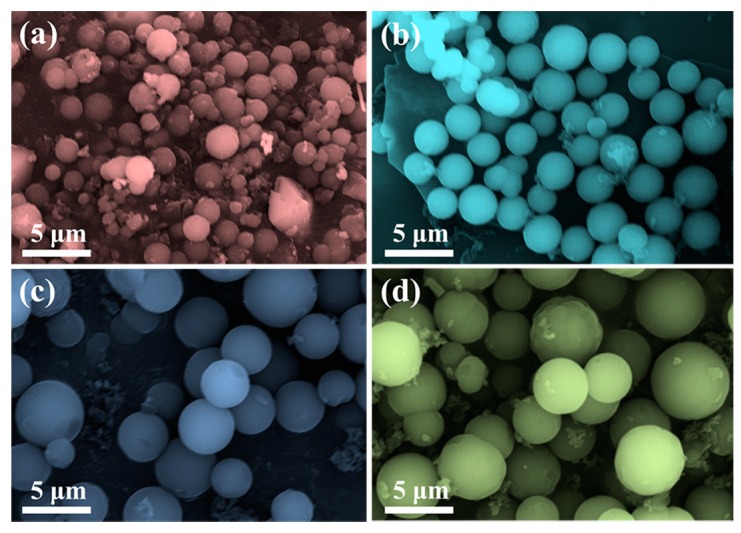
SEM images of CPCMs prepared at (**a**) 140 °C; (**b**) 160 °C; (**c**) 180 °C; and (**d**) 200 °C.

Scanning electron microscope (SEM) images of CPCMs prepared at various temperatures were shown in Figure 1a–d. CPCMs with smooth outer surfaces and spherical shapes could be found. Moreover, the diameter of CPCMs was tunable and increased from 1 μm to 5 μm as the temperature increased from 140 °C to 200 °C. The nitrogen adsorption-desorption isotherms (Figure S1a) indicated that CPCMs had a specific surface area of 5.22 m^2^ g^−1^, which was in agreement with the carbon microspheres prepared from glucose and pentosan [13].

**Figure 2:**
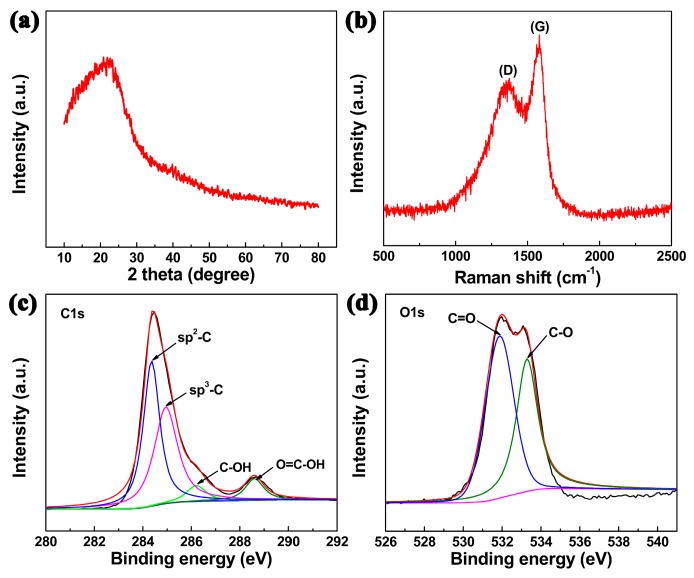
(**a**) XRD pattern, (**b**) Raman spectrum, and (**c**,**d**) high-resolution XPS spectra of C1s and O1s of CPCMs.

To analyze the phase composition of CPCMs, X-ray diffraction (XRD) was performed as shown in Figure 2a. The broad peak at about 2θ = 22° was attributed to the amorphous carbon [14]. A Raman spectrum was used to study the state of carbon. As presented in Figure 2b, the two peaks at 1577 cm^−1^ (G-band) and 1383 cm^−1^ (D-band) were associated with sp^2^ and sp^3^ hybridized carbon [10] in the CPCMs, respectively. Fourier transform-infrared (FTIR) spectra of CPCMs, as in Figure S1b, showed that the bands at 1620, 1700, 2930, and 3440 cm^−1^ corresponded to C=C, C=O, C-H, and O-H, respectively [15]. X-ray photoelectron spectra (XPS) were also employed to investigate the chemical composition of CPCMs. The high-resolution of C1s spectrum was decomposed into four peaks at 284.5, 285.0, 286.2, and 288.6 eV (Figure 2c) assigned to sp^2^-C, sp^3^-C, C-OH and O=C-OH groups [16], respectively. Additionally, the O1s spectrum could be resolved into two peaks at 533.0 (C-O) and 531.6 (C=O) eV (Figure 2d) [3]. XPS and FTIR data suggested that the abundant -COOH existed in CPCMs, which likely played important roles in the MB adsorption process owing to their electrostatic interaction.

**Figure 3:**
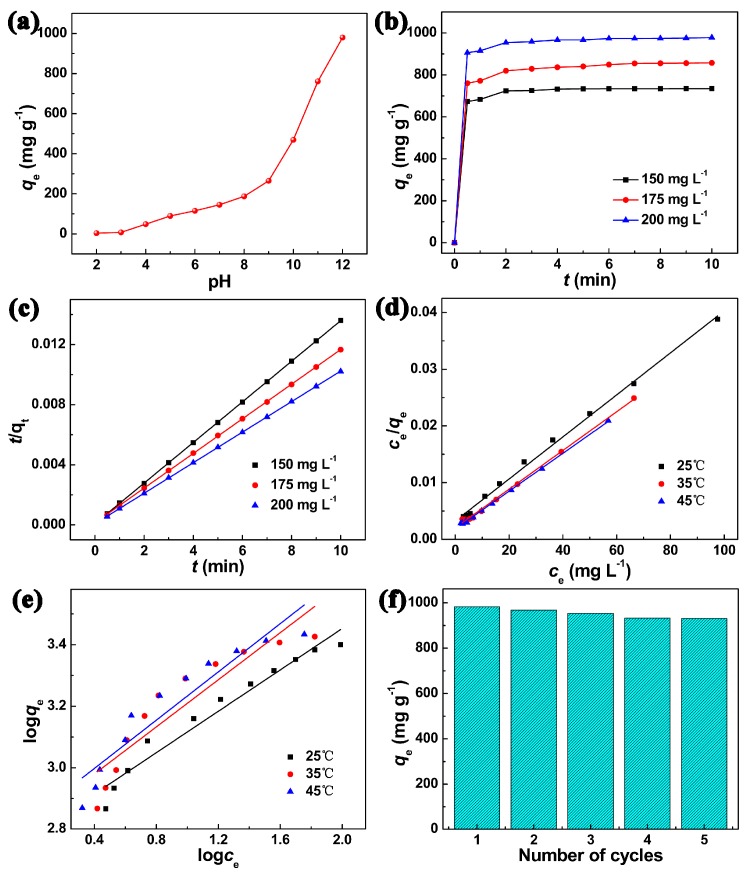
Effect of (**a**) pH (*c*_0_: 200 mg L^−1^, 2 h, 25 °C), (**b**) contact time (*c*_0_: 150, 175 and 200 mg L^−1^, pH: 12, 25 °C), (**c**) pseudo-second-order kinetics, and (**d**–**e**) Langmuir and Freundlich isotherms of MB adsorption on CPCMs; (**f**) The reusability of CPCMs toward MB adsorption (*c*_0_: 200 mg L^−1^, pH: 12).

Interestingly, CPCMs obtained at 200 °C exhibited a higher equilibrium adsorption capacity (*q*_e_) of MB than those of the materials prepared at 140 °C, 160 °C, and 180 °C (Figure S2a), and was therefore chosen for the following experiments. Fundamentally, pH is a significant parameter for MB adsorption [17]. Clearly, with the aqueous pH increased from 2 to 12, *q*_e_ improved significantly from 3.7 to 979.3 mg g^−1^ (Figure 3a). MB is a cationic dye attributed to its positively charged amine group [18], while CPCMs are negatively charged due to their surface -COOH. Therefore, at the lower pH, the decreased *q*_e_ was due to the protons in competition with MB molecules for the adsorption sites [19]. With an increase in pH, the electrostatic interaction between MB molecules and CPCMs enhanced, leading to a higher *q*_e_. It is noteworthy that the adsorption process was extremely rapid within the first 0.5 min (adsorption capacity up to 905.8 mg g^−1^ at *t* = 0.5 min, *c*_0_ = 200 mg L^−1^, Figure 3b) owing to the greater availability of vacant adsorption sites, and then slowed gradually due to the repulsive forces between the free MB molecules in the bulk phase and the adsorbed MB molecules on CPCMs [20]. Subsequently, the adsorption reached equilibrium at about 5 min. The *q*_e_ increased with the raised *c*_0_ (Figure S2b), revealing the favorable adsorption at the higher *c*_0_, which provided a vital driving force to overcome the mass transfer resistance of MB molecules between the aqueous phase and the solid phase [21]. However, *q*_e_ decreased a little in various NaCl concentrations from 0.1 to 0.5 M because Na^+^ could occupy some adsorption sites (Figure S2c). Additionally, the increased adsorption temperature resulted in higher *q*_e_ (Figure S2d), indicating that the MB adsorption was an endothermic process [4].

The adsorption kinetics of CPCMs was studied by the pseudo-first-order and pseudo-second-order kinetics models (Figure S2e and Figure 3c). As shown in Table S1, all the correlation coefficients (*R*^2^) of the pseudo-second-order model were similar and higher than 0.999, which were larger than those of the pseudo-first-order model (≤0.9824), indicating all the kinetics data fitted well with the pseudo-second-order model. Moreover, the calculated *q*_e_ (*q*_e,cal_) from the pseudo-second-order model was consistent with the experimental *q*_e_ (*q*_e,exp_), further revealing that the adsorption process was more suitable to be depicted by the pseudo-second-order model [14]. The Langmuir and Freundlich isotherms were used to describe the essence of solute-surface interaction between MB molecules and CPCMs, as well as to quantitatively analyze the adsorption capacity of CPCMs. As shown in Figure 3d,e and Table S2, the *R*^2^ from the Langmuir isotherm (≥0.9967) was higher than those of the Freundlich isotherm (≤0.9554), indicating that the Langmuir isotherm fitted better to the experimental data [22]. This result suggested that the MB adsorption on CPCMs was a monolayer adsorption that occurred on the heterogeneous surface of CPCMs, which was in good agreement with other reported absorbents [3,19]. From the Langmuir isotherm equation, the calculated maximum monolayer adsorption capacity (*q*_m_) was 2697.5 at 25 °C, which was significant higher than those of other reported adsorbents (Table S3). The reusability of the adsorbent is also an important parameter for practical application. It was found that the *q*_e_ decreased slightly after five cycles from an initial value of 982.9 to 931.4 mg g^−1^ (Figure 3f), indicating that CPCMs exhibited good reusability for MB adsorption. The good reusability of CPCMs could be attributed to their stable structures even after five cycles (Figure S2f) [3].

**Figure 4:**
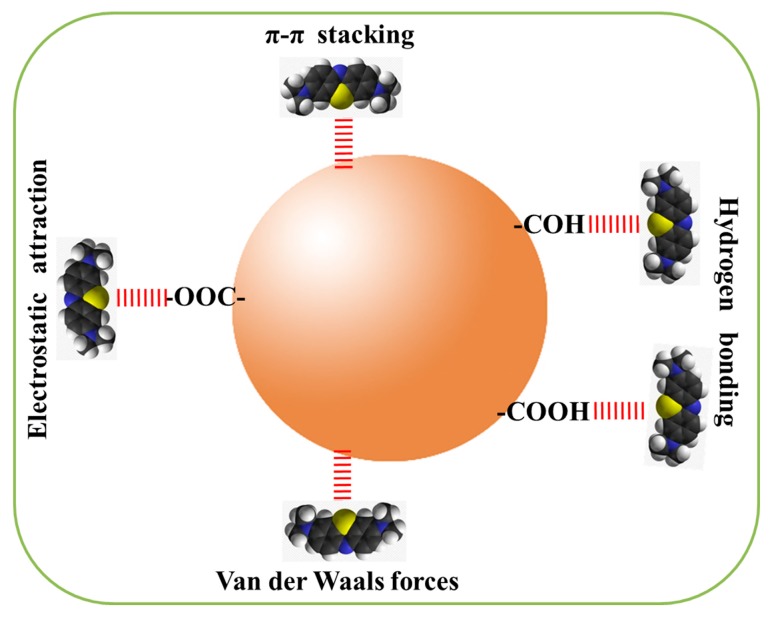
A potential mechanism for MB adsorption on CPCMs.

Based on the adsorption behavior and the chemical structures of CPCMs and MB molecules, a potential adsorption mechanism is proposed in Figure 4. Fundamentally, adsorption is a physicochemical process involving the interactions between dye molecules and an adsorbent [19]. The abundant -COOH on the surface of CPCMs and the results of the pH effect on the adsorption capacity indicated that the electrostatic interaction between CPCMs and MB molecules was mainly responsible for the adsorption mechanism. Additionally, the oxygen-containing groups such as -COOH and -COH might also adsorb some MB molecules via hydrogen bonding. As it is well known, MB is a planar molecule with an aromatic ring, while the π electrons in CPCMs are able to form the π-π interaction with MB molecules [3,4]. Moreover, van der Waals forces are also involved in the adsorption behavior [4]. These interactions likely played critical roles in the superior adsorption capacity and high adsorption rate of CPCMs. Therefore, the adsorption enhancement mechanism of CPCMs could be attributed to the strong interactions between MB molecules and CPCMs.

## 3. Conclusions

In this work, citrus pectin-derived, green, carbon microspheres were prepared successfully by a facile hydrothermal method without any hazardous chemicals. The obtained CPCMs had a superior adsorption capacity, high adsorption rate, and good reusability toward MB adsorption. The kinetics and isotherm data were well depicted by the pseudo-second-order kinetics model and the Langmuir isotherm, respectively. The adsorption enhancement mechanism of CPCMs was suggested to be the effect of interactions between CPCMs and MB molecules. The as-prepared CPCMs can be employed as a potential absorbent for the removal of MB from wastewater.

## Supplementary Materials

The following are available online at http://www.mdpi.com/2079-4991/7/7/161/s1.
