# Supplementary material for: Citrus Pectin-Derived Carbon Microspheres with Superior Adsorption Ability for Methylene Blue
Source: Nanomaterials (Basel). 2017 Jun 30;7(7):161. doi: 10.3390/nano7070161 (PMC5535227; doi:10.3390/nano7070161)
Supplement: Supplementary file 1 [file nanomaterials-07-00161-s001.pdf]

Supplementary Material

Citrus Pectin-Derived Carbon Microspheres with Superior Adsorption Ability for Methylene Blue

Wenlin Zhang[1,2] and Zhiqin Zhou[1,3*]

[1] *College of Horticulture and Landscape Architecture, Southwest University, Chongqing 400716, China;*

[2] *Institute of Special Plants, Chongqing University of Arts and Sciences, Yongchuan 402160, China;*

[3] *Key Laboratory of Horticulture Science for Southern Mountainous Regions, Ministry of Education, Chongqing 400715, China.*

Supplementary caption:

Figure S1. (a) Nitrogen adsorption-desorption isotherm of CPCMs; (b) FTIR spectra of citrus pectin and CPCMs.

Figure S2. Effect of (a) preparation temperature (c_0: 200 mg L^{-1}, 25℃, 2 h, pH: 12), (b) c_0 (25℃, 2 h, pH: 12), (c) ionic strength (c_0: 200 mg L^{-1}, 25℃, 2 h, pH: 12), (d) adsorption temperature (c_0: 300 mg L^{-1}, 2 h, pH: 12), and (e) pseudo-first-order kinetics model of MB adsorption on CPCMs; (f) SEM image of CPCMs after five cycles of adsorption-desorption.

Table S1. Kinetics model parameters of MB adsorption on CPCMs.

Table S2. Isotherm model parameters of MB adsorption on CPCMs.

Table S3. Comparison of the maximum adsorption capacities of MB on various adsorbents.

Materials and Methods

Materials

Citrus pectin (Galacturonic acid ≥74.0%, dried basis) and methylene blue (MB) were purchased from Sigma-Aldrich Co. LLC. (St Louis, USA). Other chemicals were obtained from Chengdu Kelong Chemical Reagent Co. (Sichuan, China). Ultrapure water was used for the experiments.

Instruments

The morphology of prepared materials was investigated by a JSM-6510LV scanning electron microscope (SEM, Japan) operated at 20 kV. X-ray diffraction (XRD) patterns were obtained by a Shimadzu XRD-7000 diffractometer. Fourier transform-infrared (FTIR) spectra, X-ray photoelectron spectra (XPS), and UV-vis absorption spectra were recorded using a Nicolet 6700 spectrophotometer, a Thermo Fisher Scientific Escalab 250Xi photoelectron spectrometer, and a Perkin Elmer Lambda 25 spectrophotometer, respectively. Nitrogen adsorption-desorption isotherm was measured by a Micromeritics ASAP 2020 V4.00 volumetric adsorption analyzer.

Synthesis of Citrus Pectin -Derived Carbon Microspheres (CPCMs)

CPCMs was prepared by a facile hydrothermal method using citrus pectin as the carbon source. In a typical process, 1.0 g citrus pectin was dissolved into 40 ml ultrapure water under vigorous stirring for 1 h at 25 ℃. Subsequently, the prepared pectin solution was transferred into a 50 mL

*Corresponding author. E-mail: zhouzhiqin@swu.edu.cn

Teflon-lined stainless steel autoclave and heated at 140-200 °C for 12 h. After the autoclave cooled to room temperature, the product was collected by centrifugation at 8000 rpm and washed with ultrapure water and ethanol, and then dried at 60 °C.

Batch Adsorption Experiments

Batch adsorption experiments were carried out in glass bottles of 20 mL containing 2 mg of CPCMs and 10 mL of MB aqueous solutions with the initial concentrations ranging from 150 to 600 mg L^{-1}. Subsequently, the mixtures were shaken in a shaking incubator for different times at 200 rpm. Then, the mixtures were centrifuged at 8000 rpm and the supernatant concentrations of MB were determined by a UV-Vis spectrophotometer at 664 nm. The effect of the solution pH on MB adsorption on CPCMs was investigated by varying the pH from 2 to 12. The effect of temperature on MB adsorption was also studied by keeping the temperature at 25-45℃. The adsorption capacity of MB by CPCMs was calculated via the following equation:

$$q_{\mathrm{e}} = \frac{(c_0 - c_{\mathrm{e}})V}{m} \tag{1}$$

q_{e} (mg g^{-1}) represents the equilibrium adsorption capacity of MB on CPCMs, c_0 (mg L^{-1}) and c_{e} (mg L^{-1}) are the initial and equilibrium concentrations of MB, respectively. V (L) is the volume of the solution, and m (g) is the mass of CPCMs.

Adsorption Kinetic and Isotherm Models

To understand the adsorption dynamics of the MB-CPCMs system in relation to time and to depict the nature of solute-surface interactions between CPCMs and MB, as well as to investigate the performance of CPCMs, two kinetic models (pseudo-first-order and pseudo-second-order) and two isotherm models (Langmuir and Freundlich) were studied. The equations are listed as follows:

$$\log(q_{\mathrm{e}} - q_{\mathrm{t}}) = \log q_{\mathrm{e}} - \frac{k_1 t}{2.303} \tag{2}$$

$$\frac{t}{q_{\mathrm{t}}} = \frac{1}{k_2 q_{\mathrm{e}}^2} + \frac{t}{q_{\mathrm{e}}} \tag{3}$$

$$\frac{c_{\mathrm{e}}}{q_{\mathrm{e}}} = \frac{c_{\mathrm{e}}}{q_{\mathrm{m}}} + \frac{1}{b q_{\mathrm{m}}} \tag{4}$$

$$\log q_{\mathrm{e}} = \log k + \frac{1}{n}\log c_{\mathrm{e}} \tag{5}$$

q_{t} (mg g^{-1}) represents the adsorption capacity of MB on CPCMs at any time t (min), k_1(min^{-1}) and k_2 (g mg^{-1} min^{-1}) are the pseudo-first-order and pseudo-second-order adsorption rate constants, respectively, and t is the contact time (min). q_{m} (mg g^{-1}) is the maximum adsorption capacity of MB on CPCMs, b (L mg^{-1}) is the Langmuir adsorption constant, k is the indicator of adsorption capacity, and $1/n$ represents the heterogeneity factor.

Regeneration Tests

For the regeneration study, 2 mg of CPCMs were added to 10 mL of MB solution (200 mg L^{-1}) at pH 12 and the mixture was shaken at 200 rpm for 10 min at 25℃. After adsorption and centrifugation, the supernatant MB solution was discarded, leaving CPCMs. Ethanol is one kind of common desorption agent for adsorbent regeneration in dye wastewater treatment [1]. Here, ethanol was used as a desorption agent to remove MB molecules adsorbed on the CPCMs. Then,

the MB-adsorbed CPCMs were added to 10 mL of ethanol with pH 2 adjusted by 0.1 M HCl [2] and sonicated for 2 min. Subsequently, the CPCMs were isolated from the solution by centrifugation and used for the next cycle. The final concentrations of MB were determined by UV-Vis spectra. The adsorption-desorption processes as described were carried out successively for five times.

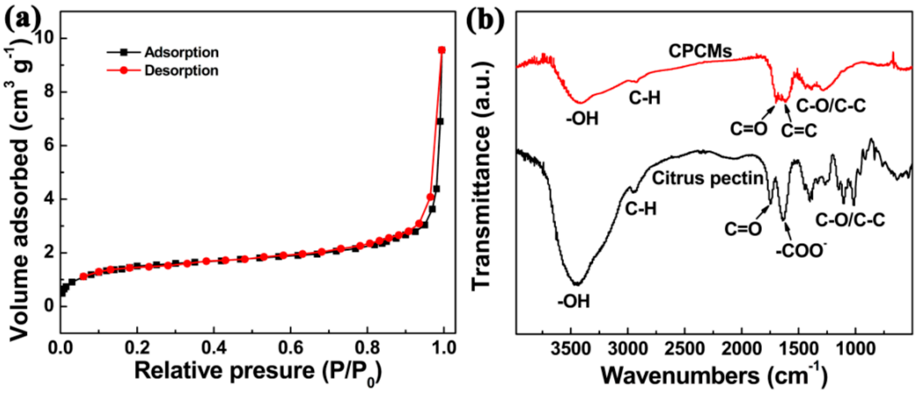

Figure S1. (a) Nitrogen adsorption-desorption isotherm of CPCMs; (b) FTIR spectra of citrus pectin and CPCMs.

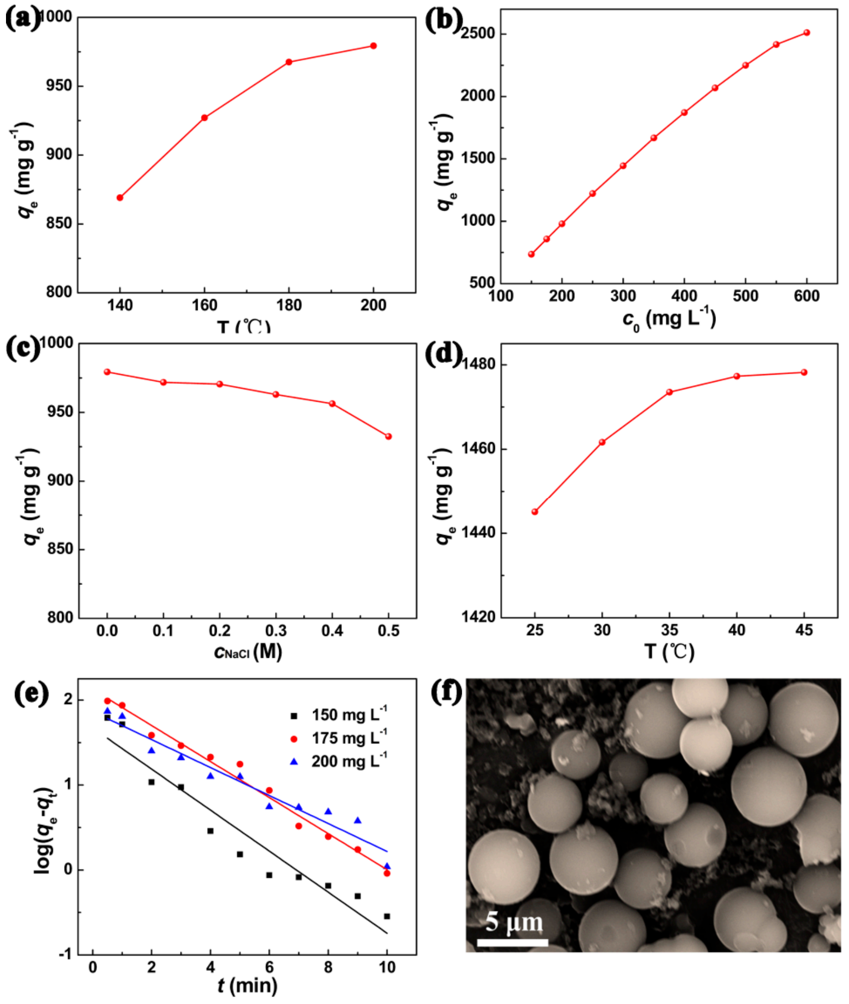

Figure S2. Effect of (a) preparation temperature (c_0: 200 mg L^{-1}, 25℃, 2 h, pH: 12), (b) c_0 (25℃,

2 h, pH: 12), (c) ionic strength (c_0: 200 mg L^{-1}, 25℃, 2 h, pH: 12), (d) adsorption temperature (c_0: 300 mg L^{-1}, 2 h, pH: 12), and (e) pseudo-first-order kinetics model of MB adsorption on CPCMs; (f) SEM image of CPCMs after five cycles of adsorption-desorption.

Table S1.

Kinetics model parameters of MB adsorption on CPCMs.

Pseudo-first-order kinetics					Pseudo-second-order kinetics		
c_0 (mg L^{-1})	$q_{e,exp}$ (mg g^{-1})	$q_{e,cal}$ (mg g^{-1})	k_1 (min^{-1})	R^2	$q_{e,cal}$ (mg g^{-1})	k_2 (g mg^{-1} min^{-1})	R^2
150	735.1	46.8	0.56	0.9209	740.7	0.026	1.0000
175	858.2	133.3	0.49	0.9824	869.6	0.020	0.9999
200	979.3	73.1	0.38	0.9408	980.4	0.016	1.0000

Table S2.

Isotherm model parameters of MB adsorption on CPCMs.

Temperature (℃)	Langmuir			Freundlich		
	q_m (mg g^{-1})	b (L mg^{-1})	R^2	k	$1/n$	R^2
25	2697.5	0.11	0.9967	602.3	0.3365	0.9554
35	2929.6	0.17	0.9971	670.3	0.3831	0.8354
45	2997.8	0.18	0.9986	692.9	0.3925	0.8823

Table S3.

Comparison of the max adsorption capacities of MB on various adsorbents.

Adsorbents	q_m (mg g^{-1})	References
Graphene/magnetite composite	43.82	[3]
Rattle-type magnetic carbon nanospheres	45.15	[4]
Carbon nanotubes	64.7	[5]
Polyaniline hydrogel	71.2	[6]
Polydopamine microspheres	90.7	[7]
Mn/MCM-41	131.6	[8]
Peach gum	298	[9]
Banana peel-derived porous carbons	385.12	[10]
h-XG/SiO_2	497.5	[11]
Glucose-derived carbon nanospheres	682	[12]
**Citrus pectin-derived carbon microspheres**	**2697.5 (25℃)**	**This work**

References

[1] M.A.M. Salleh, D.K. Mahmoud, W.A.W.A. Karim, A. Idris,Cationic and anionic dye adsorption by agricultural solid wastes: a comprehensive review, Desalination 280 (2011) 1-13.

[2] C. Wang, C. Feng, Y. Gao, X. Ma, Q. Wu, Z. Wang, Preparation of a graphene-based magnetic

nanocomposite for the removal of an organic dye from aqueous solution, Chem. Eng. J. 173 (2011) 92–97.

[3] L. Ai, C. Zhang, Z. Chen, Removal of methylene blue from aqueous solution by a solvothermal-synthesized graphene/magnetite composite, J. Hazard. Mater., 192 (2011) 1515-1524.

[4] Y. Shao, L. Zhou, C. Bao, J. Ma, A facile approach to the fabrication of rattle-type magnetic carbon nanospheres for removal of methylene blue in water, Carbon, 89 (2015) 378-391.

[5] Y. Yao, F. Xu, M. Chen, Z. Xu, Z. Zhu, Adsorption behavior of methylene blue on carbon nanotubes, Bioresour. Technol., 101 (2010) 3040-3046.

[6] B. Yan, Z. Chen, L. Cai, Z. Chen, J. Fu, Q. Xu, Fabrication of polyaniline hydrogel: Synthesis, characterization and adsorption of methylene blue, Appl. Surf. Sci., 356 (2015) 39-47.

[7] J. Fu, Z. Chen, M. Wang, S. Liu, J. Zhang, J. Zhang, R. Han, Q. Xu, Adsorption of methylene blue by a high-efficiency adsorbent (polydopamine microspheres): Kinetics, isotherm, thermodynamics and mechanism analysis, Chem. Eng. J., 259 (2015) 53-61.

[8] Y. Shao, X. Wang, Y. Kang, Y. Shu, Q. Sun, L. Li, Application of Mn/MCM-41 as an adsorbent to remove methyl blue from aqueous solution, J. Colloid Interface Sci., 429 (2014) 25-33.

[9] L. Zhou, J. Huang, B. He, F. Zhang, H. Li, Peach gum for efficient removal of methylene blue and methyl violet dyes from aqueous solution, Carbohydr. Polym., 101 (2014) 574-581.

[10] R. L. Liu, Y. Liu, X. Y. Zhou, Z. Q. Zhang, J. Zhang, F. Q. Dang, Biomass-derived highly porous functional carbon fabricated by using a free-standing template for efficient removal of methylene blue, Bioresour. Technol., 154 (2014) 138-147.

[11] S. Ghorai, A. Sarkar, M. Raoufi, A.B. Panda, H. Schönherr, S. Pal, Enhanced Removal of Methylene Blue and Methyl Violet Dyes from Aqueous Solution Using a Nanocomposite of Hydrolyzed Polyacrylamide Grafted Xanthan Gum and Incorporated Nanosilica, ACS Appl. Mater. Interfaces, 6 (2014) 4766-4777.

[12] X. Song, Y. Wang, K. Wang, R. Xu, Low-Cost Carbon Nanospheres for Efficient Removal of Organic Dyes from Aqueous Solutions, Ind.Eng.Chem.Res., 51 (2012) 13438-13444.
